# 高通量固相萃取-超高效液相色谱-串联质谱法测定血清中17种全氟/多氟烷基化合物

**DOI:** 10.3724/SP.J.1123.2024.03007

**Published:** 2025-03-08

**Authors:** Qiang LIN, Jian WANG, Jingjing LI, Dongxia SU, Meili LI, Jia WANG, Yumin NIU, Bing SHAO

**Affiliations:** 1.北京市延庆区疾病预防控制中心, 北京 102100; 1. Beijing Yanqing District Center for Disease Prevention and Control, Beijing 102100, China; 2.北京市延庆区永宁社区卫生服务中心, 北京 102100; 2. Yongning Community Health Service Center in Beijing Yanqing District, Beijing 102100, China; 3.北京市疾病预防控制中心, 食物中毒诊断溯源技术北京市重点实验室, 北京 100013; 3. Beijing Key Laboratory of Diagnostic and Traceability Technologies for Food Poisoning, Beijing Center for Disease Prevention and Control, Beijing 100013, China; 4.首都医科大学公共卫生学院, 北京 100069; 4. School of Public Health, Capital Medical University, Beijing 100069, China

**Keywords:** 固相萃取, 超高效液相色谱-串联质谱法, 全氟/多氟烷基化合物, 血清, solid-phase extraction (SPE), ultra-high performance liquid chromatography-tandem mass spectrometry (UHPLC-MS/MS), perfluorinated/polyfluoroalkyl compounds (PFASs), serum

## Abstract

全氟烷基和多氟烷基化合物(PFASs)因其持久性、毒性和容易富集等特点,成为国内外广泛关注的一类新污染物。开展人体内PFASs的生物监测和暴露评估对于评估这些化学物质对人体健康的风险具有重要意义。因此亟需开发准确灵敏、方便快捷的检测方法。本研究采用自制的不需要活化、平衡的直通式HMR固相萃取柱净化样本,结合超高效液相色谱-串联质谱(UHPLC-MS/MS),建立了血清中17种PFASs的检测方法。50 μL血清样本在HMR固相萃取柱中用400 μL乙腈提取(每次200 μL)并净化2次后,使用Poroshell 120 EC-C18色谱柱(100 mm×3 mm, 2.7 μm)分离,以5 mmol/L乙酸铵水溶液和甲醇为流动相进行梯度洗脱,ESI^-^离子源电离,采用多反应监测模式检测,同位素内标法定量。结果表明,17种PFASs在相应质量浓度范围内线性关系良好(*r*^2^>0.995),检出限为3.6~14 ng/L,定量限为11~42 ng/L。血清样本加标回收率为89.3%~110.2%,日内相对标准偏差(RSD)为2.5%~9.8%,日间RSD为3.6%~10.2%。将方法应用于20份人体血清样品的检测,结果显示17种化合物均有检出。本方法仅需50 μL血清样品,可以在HMR固相萃取柱中一步实现提取和净化,且固相萃取过程可以实现与96孔板的适配。方法操作简便,灵敏度高,样本用量少,检测效率高,适用于人群血清样本中PFASs的大规模监测及暴露评估。

## Abstract (en)

Perfluorinated/polyfluoroalkyl compounds (PFASs) are a group of synthetic chemicals. Since the 1940s, PFASs have been widely used in industrial and daily consumer fields. PFASs can enter the human body through various pathways, such as drinking water, food, and air. Toxicological studies have shown that PFASs feature developmental and reproductive toxicity. Thus, the accurate assessment of PFAS exposure levels plays an important role in determining the health risks of these compounds. Biological monitoring is considered the most ideal means of monitoring and evaluating PFAS levels in the human body. The concentration of PFASs in serum can directly reflect their level of absorption in the human body after exposure; therefore, serum is widely used as a biological matrix for evaluating PFASs.

In this study, a high-throughput solid-phase extraction (SPE)-ultra-high performance liquid chromatography-tandem mass spectrometry (UHPLC-MS/MS) was developed for the determination of 17 PFASs in serum. A laboratory-made straight-through SPE column was used to purify serum samples. The SPE column used a new inorganic material with a low perfluorinated compound background. Unlike traditional SPE columns, the extraction column does not require activation before use, and the sample can be directly loaded into it for purification. The straight-through SPE column adopts a stepped design, which does not require a large sample volume and is suitable for the pretreatment of precious biological samples such as serum. The serum sample size adopted in this study was only 50 μL, which is much lower than that required by other extraction methods. The straight-through SPE column can be used in conjunction with a 96-well plate, and the purification treatment of 96 serum samples can be completed within 30 min with high efficiency.

The mass spectrometry parameters were optimized, and the results indicated that the electrospray ionization source temperature had a significant impact on the PFASs with later elution peaks. The effects of ion-source temperatures of 400 and 500 ℃ on the peak shapes and response values of the target compounds were compared, and 400 ℃ was selected. The sample pretreatment and ultra-high performance liquid chromatographic conditions were optimized. Briefly, 50 μL of the sample was loaded into the SPE column and extracted twice using 200 μL of acetonitrile each time. The purified solution was collected, blown with nitrogen at 40 ℃ to near dryness, dissolved in a 50% methanol aqueous solution, and then separated on a Poroshell 120 EC-C18 chromatographic column (100 mm×3 mm, 2.7 μm) with 5 mmol/L ammonium acetate aqueous solution and methanol as mobile phases. Detection was conducted in multiple-reaction monitoring mode, and quantification was performed using an isotope internal standard method. The 17 detected PFASs had good linear relationships within the corresponding mass concentration ranges (*r*^2^>0.995), with limits of detection ranging from 3.6 ng/L to 14 ng/L and limits of quantification ranging from 11 ng/L to 42 ng/L. The recoveries in spiked serum samples were 89.3%-110.2%, the intra-day RSDs were 2.5%-9.8%, and the inter-day RSDs were 3.6%-10.2%. The method was applied to the detection of 20 human serum samples, and all 17 compounds were successfully detected. The proposed method is easy to operate, highly sensitive, has a small sample size and high detection efficiency, and is suitable for the large-scale monitoring and exposure assessment of PFASs in human serum samples.

全氟/多氟烷基化合物(PFASs)是一类人类合成的化学品,因氟原子取代化合物碳链上全部或部分氢原子而得名^[1]^。自20世纪40年代以来,PFASs在工业和日常消费领域得到了广泛使用和推广,如不粘炊具、食品包装、防水材料、化妆品和消防泡沫等^[2]^。PFASs的碳-氟键使其具有高度的抗降解性,因此可以在环境中持久存在。目前在土壤、水、大气、灰尘以及食品等介质中均可检测出PFASs^[3⇓⇓-6]^。PFASs可以通过饮水、食物、空气等多种途径进入人体^[7,8]^。毒理学研究显示PFASs具有发育毒性和生殖毒性^[9]^,可造成肝损伤和慢性肾脏疾病^[10,11]^。人体生物监测被认为是最理想的监测和评估手段。血清中PFASs的浓度水平可以直接反映人体暴露后吸收的水平,因此血清被广泛用作评价PFASs的生物监测基质^[12,13]^。美国疾病预防控制中心国家健康和营养调查(NHANES)监测结果表明,在95%的人群血清样品中均检测出PFASs^[12]^。

血清样品基质复杂,残留的PFASs处于痕量水平,高效的前处理方法和灵敏的检测技术是监测血清中PFASs的关键。PFASs的检测方法有气相色谱-质谱法(GC-MS)和液相色谱-质谱法(LC-MS)^[14⇓-16]^。全氟酰胺类化合物等挥发性较强的PFASs可以直接采用GC-MS进行测定,而对于饱和蒸气压低且难挥发的PFASs,在采用GC-MS测定之前需要对其进行衍生化处理^[17]^,操作复杂,且衍生化试剂一般具有毒性和腐蚀性等危害^[18]^;LC-MS具有灵敏度高、选择性好等优点,并且无需衍生化,成为PFASs检测的主流方法。目前文献报道的血液中PFASs的前处理方法主要采用离子对液液萃取^[19,20]^、固相萃取^[21⇓-23]^、基质分散固相萃取^[24]^等。谢琳娜等^[19]^、王梓皓等^[20]^使用四丁基硫酸氢铵为离子对试剂,进一步用甲基叔丁基醚作为萃取液,采用液液萃取对200 μL血清样本进行净化处理,建立了血清样品中18种PFASs的检测方法。杨觅等^[22]^使用乙腈沉淀蛋白质,结合需要提前活化、平衡的WAX固相萃取柱净化,建立了血清中12种PFASs的检测方法。以上前处理方法存在样品取样量大、操作复杂、有机试剂使用量大、易引入污染等缺点。

本研究根据美国NHANES生物监测结果^[9]^,同时结合毒理特性,选取了全氟辛烷磺酸(PFOS)、全氟辛酸(PFOA)等检出率较高且毒性相对较强的17种PFASs为研究对象。采用直通式HMR固相萃取柱净化血清样本,该固相萃取柱使用新型无机材料,无需经过传统固相萃取柱的活化、淋洗、洗脱等流程,可直接上样,在萃取柱内即可完成沉淀蛋白质、去除磷脂类化合物等流程。直通式固相萃取柱采用阶梯式设计,净化效率高,无需大体积样本上样,适用于血清等特别珍贵的生物样本的前处理。本研究血清的上样量仅为50 μL,远低于其他研究方法。该固相萃取柱和96孔板配套使用,30 min即可完成96个血清样本的净化处理,适用于生物监测中样本的大批量检测。综上,本研究使用直通式固相萃取柱净化样本,结合超高效液相色谱-串联质谱建立了血清中17种PFASs的检测方法。

## 1 实验部分

### 1.1 仪器与试剂

SCIEX Triple Quad^TM^ 6500+超高效液相色谱-三重四极杆质谱仪(美国SCIEX公司); MixMate 96孔涡旋混匀仪(美国Eppendorf公司);固相萃取正压装置(美国Waters公司); Milli-Q纯水系统(美国Millipore公司)。甲醇、乙腈(色谱纯,德国Merck公司);甲酸(色谱纯,美国Sigma-Aldrich公司); 自制HMR固相萃取柱(填料10 mg)。

标准品:全氟丁酸(PFBA)、全氟戊酸(PFPeA)、全氟己酸(PFHxA)、全氟庚酸(PFHpA)、PFOA、全氟壬酸(PFNA)、全氟癸酸(PFDA)、全氟十一酸(PFUnDA)、全氟十二酸(PFDoDA)、全氟十三酸(PFTriDA)、全氟十四酸(PFTeDA)、全氟丁烷磺酸(PFBS)、全氟己烷磺酸(PFHxS)、全氟庚烷磺酸(PFHpS)、PFOS、6∶2氯代多氟烷基醚磺酸(6∶2 Cl-PFESA)、8∶2氯代多氟烷基醚磺酸(8∶2 Cl-PFESA)和12种同位素内标^13^C_4_-PFBA、^13^C_5_-PFPeA、^13^C_5_-PFHxA、^13^C_4_-PFHpA、^13^C_8_-PFOA、^13^C_9_-PFNA、^13^C_6_-PFDA、^13^C_7_-PFUnDA、^13^C_2_-PFTeDA、^13^C_3_-PFBS、^13^C_3_-PFHxS、^13^C_8_-PFOS的质量浓度均为2.0 mg/L,购于加拿大Wellington Laboratories公司。

血清样品来源:血清样本于2016年采自北京大兴区和密云区,已通过北京市疾病预防控制中心伦理委员会批准,编号:BJCDC/GD12-KJ-F03。

### 1.2 标准溶液的配制

#### 1.2.1 标准储备溶液

分别准确吸取PFBA标准溶液1.25 mL,其余16种标准溶液各0.5 mL于10 mL容量瓶中,加入甲醇定容至刻度线,配制成PFBA质量浓度为250 μg/L,其余16种化合物质量浓度为100 μg/L的混合标准溶液,于-20 ℃保存待用。

#### 1.2.2 混合内标工作溶液

分别准确吸取12种同位素内标适量,使用甲醇逐级稀释,得到质量浓度分别为100 μg/L和2.5 μg/L的PFASs内标工作溶液,100 μg/L内标工作溶液用于配制标准工作溶液,2.5 μg/L内标工作溶液用于样品前处理。于-20 ℃保存待用。

#### 1.2.3 系列标准工作溶液

使用甲醇对混合标准溶液和100 μg/L内标工作溶液进行稀释,配制成PFBA质量浓度为0.05、0.10、0.20、0.50、1.0、2.0、5.0、10.0、20.0 μg/L,其余16种化合物质量浓度为0.02、0.05、0.10、0.20、0.50、1.0、2.0、5.0、10.0、20.0 μg/L的系列标准工作溶液。标准工作溶液中同位素内标质量浓度为2.5 μg/L。

### 1.3 样品前处理

准确吸取50 μL血清至HMR固相萃取小柱中,加入50 μL 2.5 μg/L的内标工作溶液,沿萃取柱内壁缓慢加入200 μL乙腈,通风柜内放置5 min,待溶液通过筛板1进入填料中(图1)。将固相萃取小柱转移至正压装置上,逐步施加压力,将小柱中的液体完全缓慢排出,液体收集至96孔板中。从正压装置上取下固相萃取小柱,加入200 μL乙腈,重复提取操作一次。收集两次流出液,40 ℃氮气吹至近干,加入50 μL 50%(v/v)甲醇水溶液复溶,放置于96孔板涡旋混匀仪混匀1 min,直接进行检测。

**图1:**
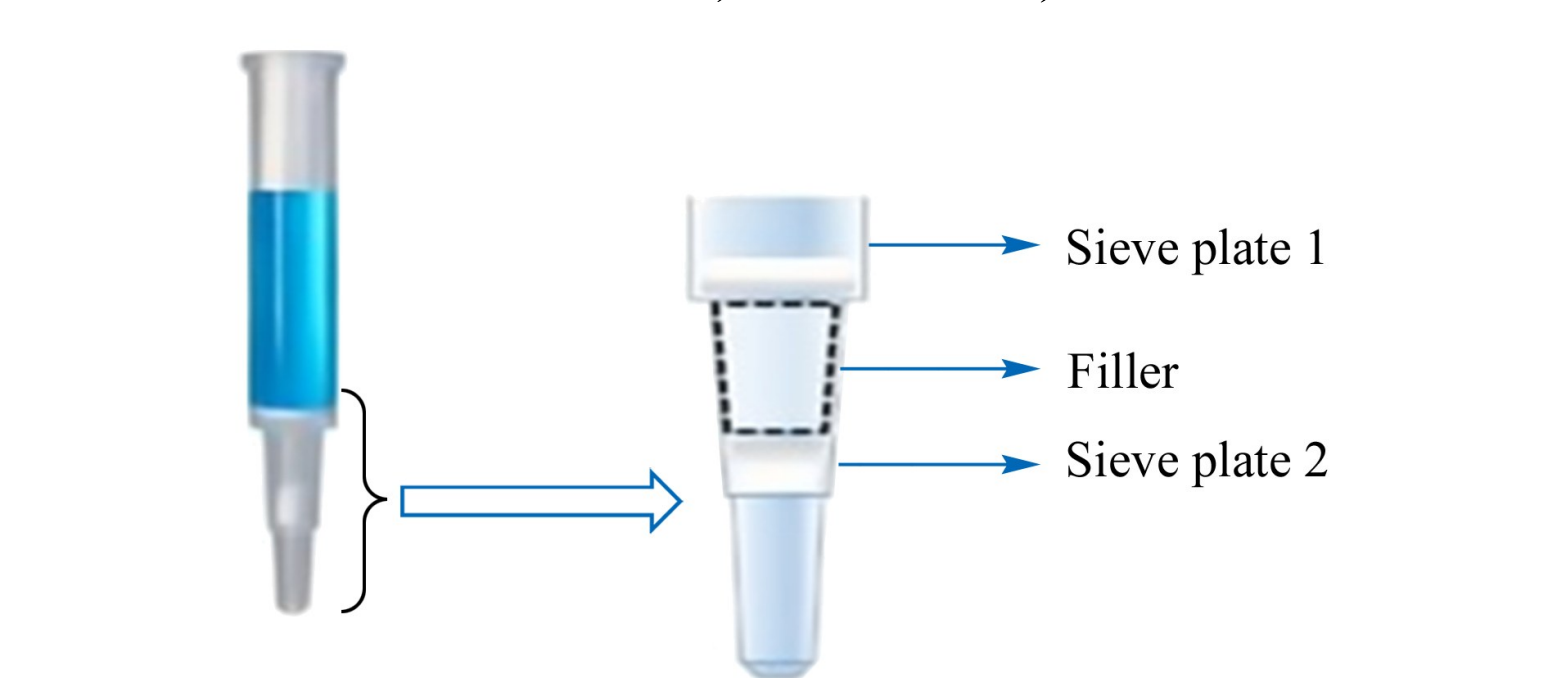
HMR固相萃取柱局部示意图

### 1.4 仪器条件

#### 1.4.1 液相色谱条件

色谱柱:Poroshell 120 EC-C18 (100 mm×3 mm, 2.7 μm,美国Agilent公司);流动相:A为5 mmol/L乙酸铵水溶液,B为甲醇;梯度洗脱程序:0~6.0 min, 40%B~95%B; 6.0~8.0 min, 95%B; 8.0~8.1 min, 95%B~40%B; 8.1~10.0 min, 40%B;流速:0.40 mL/min;柱温:40 ℃;进样体积:5 μL。

#### 1.4.2 质谱条件

电喷雾离子源,负离子模式(ESI^-^);离子源温度:400 ℃;喷雾电压:4500 V;气帘气压力:0.24 MPa;雾化气压力(GS1)和辅助加热器压力(GS2): 0.38 MPa。多反应监测(MRM)模式采集。其余质谱参数见表1。

**表1:** PFASs和同位素内标的保留时间和质谱参数

Compound	Abb.	t_R_/min	Transition ions (m/z)	DP/V	CE/eV	Internal standard
Perfluorobutyric acid	PFBA	2.19	213.1>169.0^*^	-19	-13	^13^C_4_-PFBA
Perfluoropentanoic acid	PFPeA	3.23	263.1>219.0^*^	-26	-12	^13^C_5_-PFPeA
			263.1>69.0	-26	-50	
Perfluorohexanoic acid	PFHxA	4.12	313.0>269.1^*^	-11	-13	^13^C_5_-PFHxA
			313.1>119.2	-11	-26	
Perfluoroheptanoic acid	PFHpA	4.80	363.1>319.0^*^	-20	-14	^13^C_4_-PFHpA
			363.2>169.0	-20	-24	
Perfluorooctanoate acid	PFOA	5.35	413.1>369.2^*^	-40	-15	^13^C_8_-PFOA
			413.1>169.0	-40	-25	
Perfluorononanoic acid	PFNA	5.81	463.2>419.1^*^	-19	-16	^13^C_9_-PFNA
			463.1>219.1	-19	-24	
Perfluorodecanoic acid	PFDA	6.19	513.1>469.2^*^	-52	-15	^13^C_6_-PFDA
			513.2>219.2	-52	-25	
Perfluoroundecanoic acid	PFUnDA	6.51	563.1>519.2^*^	-36	-17	^13^C_7_-PFUnDA
			563.2>269.2	-36	-24	
Perfluorododecanoic acid	PFDoDA	6.78	613.1>569.2^*^	-20	-19	^13^C_2_-PFDoDA
			613.1>269.2	-20	-24	
Perfluorotridecanoic acid	PFTriDA	7.02	663.0>619.1^*^	-17	-60	^13^C_2_-PFTeDA
			663.1>169.2	-34	-60	
Perfluorotetradecanoic acid	PFTeDA	7.22	713.2>669.0^*^	-18	-30	^13^C_2_-PFTeDA
			713.1>169.0	-37	-30	
Perfluorobutanesulfonic acid	PFBS	3.37	229.1>80.1^*^	-80	-67	^13^C_3_-PFBS
			229.1>99.2	-80	-32	
Perfluorohexanesulfonic acid	PFHxS	4.83	399.1>80.2^*^	-80	-80	^13^C_3_-PFHxS
			399.1>99.0	-80	-77	
Perfluoroheptanesulfonic acid	PFHpS	5.35	449.1>80.2^*^	-80	-101	^13^C_3_-PFHxS
			449.0>99.1	-80	-97	
Perfluorooctanesulfonic acid	PFOS	5.80	499.0>80.1	-80	-85	^13^C_8_-PFOS
			499.2>99.1^*^	-80	-94	
6∶2 chlorinated polyfluorinated ether sulfonate acid	6∶2 Cl-PFESA	5.99	531.2>351.1^*^	-34	-38	^13^C_8_-PFOS
			531.2>83.0	-34	-77	
8∶2 chlorinated polyfluorinated ether sulfonate acid	8∶2 Cl-PFESA	6.62	631.1>451.2^*^	-31	-42	^13^C_8_-PFOS
			631.0>83.2	-31	-84	
^13^C_4_-PFBA		2.25	217.0>172.0	-34	-15	
^13^C_5_-PFPeA		3.30	268.2>223.0	-58	-14	
^13^C_5_-PFHxA		4.18	318.2>273.1	-71	-14	
^13^C_4_-PFHpA		4.86	367.1>322.2	-12	-15	
^13^C_8_-PFOA		5.40	421.2>376.1	-4	-17	
^13^C_9_-PFNA		5.84	472.1>427.2	-21	-16	
^13^C_6_-PFDA		6.22	519.2>474.1	-8	-19	
^13^C_7_-PFUnDA		6.54	570.2>525.1	-96	-20	
^13^C_2_-PFDoDA		6.82	615.2>570.1	-41	-19	
^13^C_3_-PFBS		3.43	302.1>80.1	-6	-72	
^13^C_3_-PFHxS		4.88	402.1>80.1	-42	-74	
^13^C_8_-PFOS		5.83	507.1>80.1	-26	-117	

* Quantitative ion pair. DP: declustering potential; CE: collision energy.

## 2 结果与讨论

### 2.1 质谱参数优化

采用直接进样方式将5.0 μg/L标准溶液注入离子源,由于PFASs结构中含有羧基或磺酸基团,在ESI离子源下容易失去H形成[M-H]^-^的母离子。因此,本研究在负离子模式下进行母离子扫描,获得目标化合物的分子离子峰。选定母离子后,进一步进行二级质谱扫描,选择丰度较高的两个特征碎片作为子离子,同时优化其碰撞电压(CE)和去簇电压(DP)。最后在MRM模式下对离子源温度、气帘气压力、GS1和GS2等参数进行优化,最终优化参数见1.4.2节和表1。对于PFBA等11种全氟羧酸类化合物,容易失去羧基(*m/z* 44),形成[M-COO-H]^-^的特征碎片,C-C键进一步断裂,形成[C_2_HF_5_]^-^(*m/z* 119)、[C_3_HF_7_]^-^(*m/z* 169)等系列全氟烷基碎片。PFBA由于仅含有3个C原子,C-C键很难发生断裂,因此仅有一个[M-COO-H]^-^的特征碎片。对于PFBS等4种全氟磺酸类化合物,容易产生磺酸基[HSO_3_]^-^特征碎片(*m/z* 80);与磺酸基相邻的C原子上的F原子发生重排,形成[SO_3_F]^-^的特征碎片(*m/z* 99)^[25]^。对于6∶2 Cl-PFESA和8∶2 Cl-PFEBA两种多氟烷基醚磺酸,C-O键易发生断裂,形成[M-C_2_F_4_SO_3_-H]^-^的特征碎片;与磺酸基相邻的C原子上的F原子发生重排,形成[SO_3_F]^-^(*m/z* 99)和[SO_2_F]^-^(*m/z* 83)的特征碎片。本研究发现离子源温度对出峰较晚的PFASs的影响较大。以PFOA为例,图2显示了离子源温度分别为500 ℃和400 ℃时PFOA的出峰情况,当离子源温度为500 ℃时(图2a),化合物峰形呈现锯齿状波动,化合物的离子化过程不稳定,且响应较低。当离子源温度为400 ℃时(图2b)时,色谱峰分叉变少,响应值高。因此选择离子源温度为400 ℃。

**图2:**
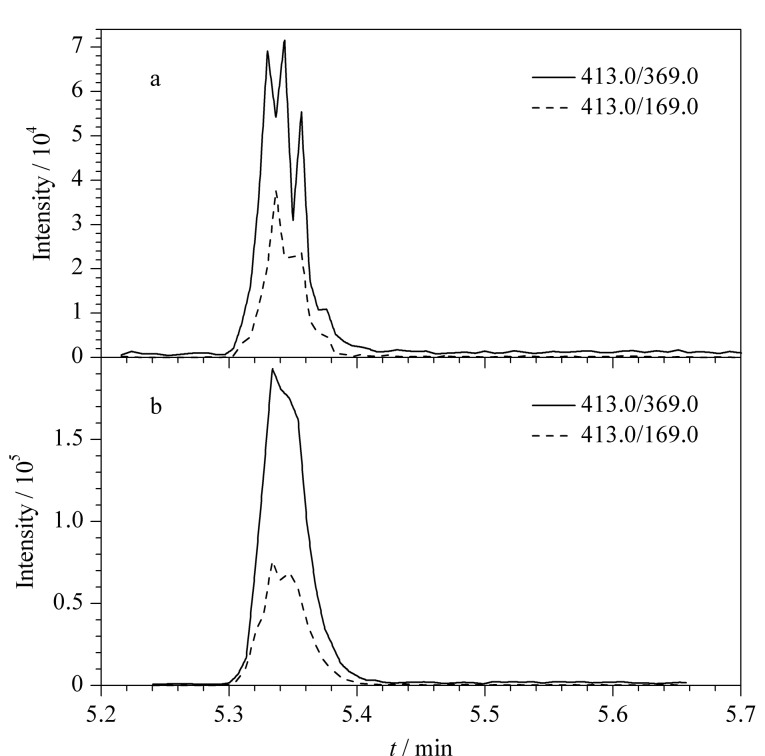
不同离子源温度对PFOA色谱峰的影响

### 2.2 色谱条件优化

文献显示,甲醇是LC-MS/MS测定PFASs常用的流动相^[16,17]^。本研究采用甲醇作为有机相,对比了4种水相体系:超纯水、1 mmol/L乙酸铵、5 mmol/L乙酸铵和10 mmol/L乙酸铵,实验结果表明,乙酸铵的加入可以显著提高目标化合物的响应值。使用5 mmol/L乙酸铵水溶液-甲醇作为流动相时,17种目标物分离良好、峰形对称、响应值最高。因此选择5 mmol/L乙酸铵水溶液-甲醇作为流动相,17种目标化合物的色谱图见图3。

**图3:**
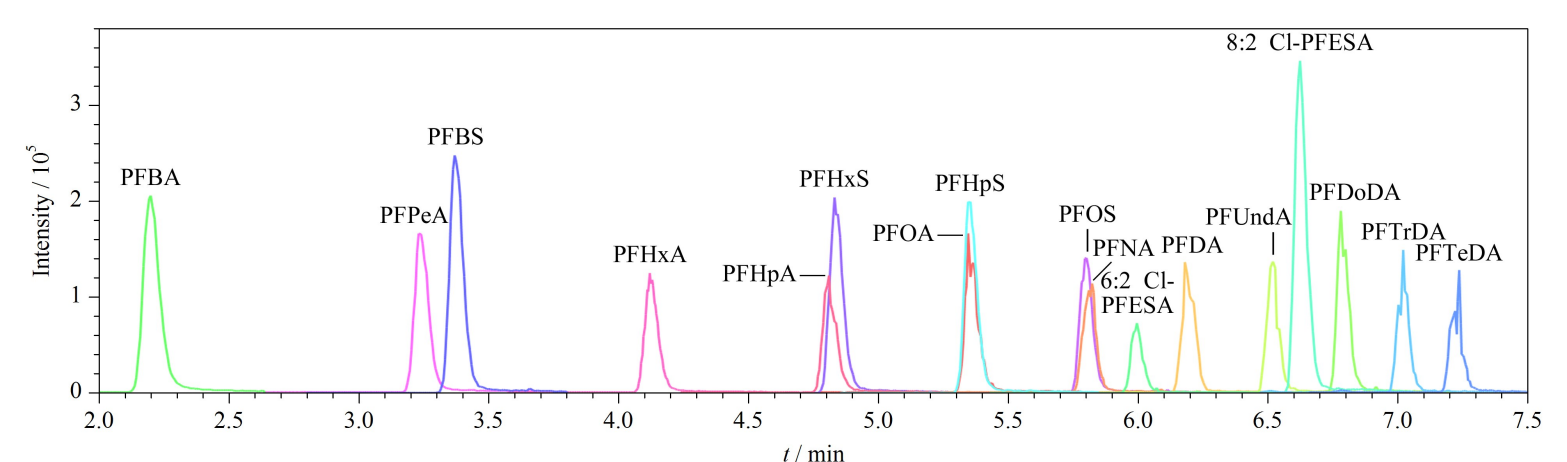
17种PFASs的总离子流图(质量浓度为10 μg/L)

### 2.3 前处理条件的优化

#### 2.3.1 提取试剂的优化

乙腈是沉淀蛋白质最常用的有机溶剂^[15⇓-17]^,因此选取乙腈作为提取试剂开展实验。本研究首先优化了乙腈提取体积:分别采用150、200和250 μL乙腈对50 μL血清样品中17种目标化合物进行提取(加标质量浓度PFBA为4.0 μg/L,其余16种目标化合物为2.0 μg/L,提取1次)。结果如图4a所示,使用150 μL乙腈对血清样本进行提取,目标化合物的绝对回收率为63.5%~71.2%,当乙腈体积为200 μL时,目标化合物的绝对回收率提升至74.0%~79.8%,进一步增加乙腈体积,目标化合物的回收率变化不大,因此选择乙腈提取体积为200 μL。

**图4:**
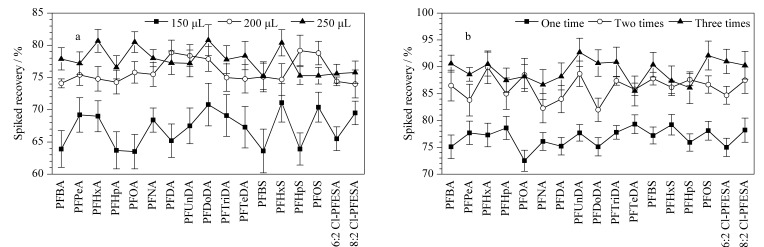
提取试剂的优化(*n*=6)

其次,考察了提取次数的影响(每次使用200 μL乙腈)。结果如图4b所示,提取1次时,目标化合物的绝对回收率为75.1%~78.7%;提取2次时,目标化合物的绝对回收率上升至84.8%~94.0%;提取3次时,目标化合物绝对回收率为85.9%~95.1%,没有明显升高。因此最终采用200 μL乙腈对血清样本进行两次提取。

#### 2.3.2 固相萃取条件的优化

血清样品富含蛋白质和磷脂,经乙腈提取后,可有效去除蛋白质。然而磷脂分子可能会抑制目标化合物的电离,并且缩短色谱柱寿命,需将血清中的磷脂分子在前处理过程中尽可能去除。HMR固相萃取柱采用了磷脂的独特吸附剂,可以有效去除磷脂。因此本研究采用HMR固相萃取柱结合96孔板进行净化。为进一步提高实验效率,我们采用一步式提取净化方式,即在固相萃取小柱上方加一个筛板(图1),在第一个筛板上完成蛋白质沉淀,提取液经过第一个筛板后,通过HMR吸附剂进行净化。将加标质量浓度PFBA为4.0 μg/L、其余16种目标化合物为2.0 μg/L的血清样品使用HMR固相萃取柱净化处理,目标物的回收率为87.0%~96.2%,相对标准偏差(RSD,*n*=6)为6.0%~10.2%,表明方法稳定可靠。

#### 2.3.3 复溶液的优化

本研究对比了不同体积分数的甲醇水溶液作为复溶液对检测结果的影响,分别使用20%、40%、50%、60%、80%的甲醇水溶液进行复溶。实验结果表明,当甲醇含量为20%、40%、50%时,17种目标物峰形良好,响应值随着甲醇含量的增高而增高。当甲醇含量大于50%时,出峰时间较早的化合物(如PFBA)受到溶剂效应的影响,色谱峰出现分叉、峰形不对称的情况。因此,选择50%甲醇水溶液作为复溶液。

### 2.4 线性关系、检出限与定量限

将系列标准工作溶液按照1.4节仪器条件进行测定。以样品中目标化合物的质量浓度为横坐标,以目标化合物的峰面积与对应同位素内标峰面积之比为纵坐标,绘制标准曲线。17种PFASs在0.02~20.0 μg/L(PFBA: 0.05~20.0 μg/L)范围内线性关系良好,相关系数(*r*^2^)均大于0. 995。以信噪比(*S/N*)=3计算检出限(LOD),以*S/N*=10计算定量限(LOQ),具体结果见表2。

**表2:** 17种PFASs的线性范围、相关系数、检出限、定量限、回收率和精密度

Compound	r^2^	LOD/(ng/L)	LOQ/(ng/L)	Recoveries/% (n=6)	RSDs/% (n=6)	
					Intra-day	Inter-day
PFBA^*^	0.9999	14	42	90.1-96.3	3.1-5.6	6.4
PFPeA	0.9989	3.6	11	89.3-97.1	5.0-5.6	6.3
PFHxA	0.9992	5.3	16	95.6-102.3	3.5-7.5	9.2
PFHpA	0.9991	4.6	14	101.2-105.1	2.5-6.1	8.6
PFOA	0.9994	4.0	12	102.5-106.2	5.1-9.8	7.7
PFNA	0.9960	5.0	15	92.1-96.3	5.0-5.6	9.6
PFDA	0.9981	4.6	14	90.5-98.7	3.0-3.6	8.8
PFUnDA	0.9982	5.0	15	107.2-109.2	3.2-7.4	5.7
PFDoDA	0.9975	4.0	12	96.5-102.3	3.5-7.6	5.6
PFTriDA	0.9991	4.3	13	93.7-96.7	6.1-8.4	10.2
PFTeDA	0.9996	5.3	16	98.4-109.3	5.6-8.5	9.6
PFBS	0.9997	5.6	17	105.5-110.2	5.6-9.4	7.8
PFHxS	0.9997	5.0	15	102.5-109.1	3.6-6.2	5.4
PFHpS	0.9994	5.0	15	96.4-106.2	3.4-5.2	3.6
PFOS	0.9992	5.3	16	94.6-103.2	3.4-5.6	8.8
6∶2 Cl-PFESA	0.9971	5.6	17	98.2-105.4	2.9-6.4	6.5
8∶2 Cl-PFESA	0.9991	6.3	19	93.5-106.7	5.2-8.4	5.4

* The linear range of PFBA is 0.05-20.0 μg/L. The linear ranges of other compounds are 0.02-20.0 μg/L.

### 2.5 准确度与精密度

在血清样本中加入低、中、高3种不同浓度的混合标准溶液,使17种PFASs的质量浓度分别为0.2、5.0、10 μg/L(PFBA质量浓度为0.5、12.5、25 μg/L),每个样本设置6个平行样本,按1.3节操作处理,分别计算17种目标化合物的加标回收率、日内RSD。连续6 d重复检测中间浓度的加标样品,计算17种目标化合物的日间RSD。17种PFASs的加标回收率为89.3%~110.2%,日内RSD为2.5%~9.8%,日间RSD为3.6%~10.2%,具体结果见表2。实验结果表明,该方法准确度高、精密度好,适合用于血清样本中17中PFASs的检测。

### 2.6 质控样品测定结果

采用国际认可的生物样品质控样SRM 1957进一步评价本方法的准确性。SRM 1957质控样采用10.7 mL超纯水复溶,准确吸取50 μL复溶后的血清样品,采用本研究所建立的方法进行测定,由于质控样的含量为μg/kg,因此按照血清密度为*ρ*=1.0^[26]^对检测结果进行换算,将检测结果与该质控样品认证的7种PFASs含量进行比较。结果如表3所示,本研究的测定结果均在含量参考值范围内,进一步证明本方法准确可靠。

**表3:** 质控样SRM 1957的测定结果(*n*=6)

Compound	Detection result/ (μg/kg)	RSD/ %	Scope of quality control samples/(μg/kg)
PFHpA	0.28	6.7	0.254-0.356
PFOA	4.96	7.8	4.56-5.44
PFNA	0.80	5.6	0.801-0.955
PFDA	0.39	4.8	0.27-0.51
PFUnDA	0.16	5.7	0.136-0.208
PFHxS	4.21	7.1	3.17-4.83
PFOS	21.25	6.3	19.8-22.4

### 2.7 实际样本的检测

采用本研究建立的方法检测20个志愿者血清样本。结果表明,17种PFASs均有检出;其中,PFOA、PFNA、PFHxS、PFHpS和PFOS的检出率为100%,质量浓度中位数分别为0.83、0.93、1.25、1.01、0.68 μg/L,范围分别为0.71~3.65 μg/L、 0.25~1.24 μg/L、0.14~1.78 μg/L、0.36~2.45μg/L和0.78~4.78 μg/L。图5为实际样品中部分化合物的色谱图。以上结果表明本研究所建立的方法可以满足人群血清样本中17种PFASs的检测。

**图5:**
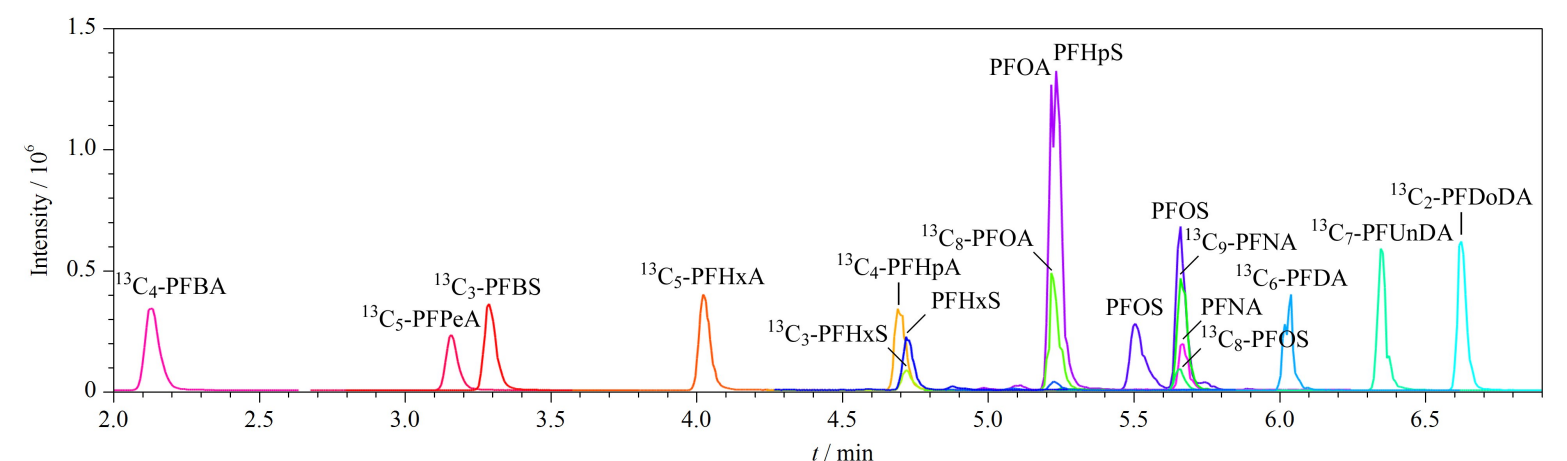
实际样品中PFHxS、PFOA、PFOS、PFNA和PFHpS的色谱图

## 3 结论

本研究建立了人体血清中17种PFASs的高通量固相萃取-超高效液相色谱-串联质谱检测方法。相比以往先提取后净化的方法,该方法可以在固相萃取柱中同时实现提取和净化,并且可以与96孔板适配,更加高效便捷,方法准确度高,血清样本用量少,适合开展人群生物样本中PFASs的大批量监测工作。
